# Organic production of vinegar from mango and papaya

**DOI:** 10.1002/fsn3.1981

**Published:** 2020-11-20

**Authors:** Koffi Maïzan Jean‐Paul Bouatenin, Kohi Alfred Kouamé, Minkapieu Edwige Gueu‐Kehi, N’Dédé Théodore Djéni, Koffi Marcellin Djè

**Affiliations:** ^1^ Department of Food Sciences and Technology Laboratory of Biotechnology and food Microbiology University Nangui Abrogoua Abidjan Côte d’Ivoire

**Keywords:** acetic fermentation, alcoholic fermentation, mango, papaya, production, vinegar

## Abstract

The study describes the transformation of mangoes of the local variety "Assabonou" and papaya solo No.8 into alcohol and then into vinegar through the process of directed fermentation. Indeed, mango and papaya juices extracted from ripe fruits contained in vials are first subjected to an alcoholic fermentation with *Saccharomyces cerevisiae* in anaerobic conditions and secondly to an acetic fermentation with strains of acetic acid bacteria cultivated from unpasteurized cider vinegar. To assess the quality of the vinegars produced, their profile and composition in organic acid and volatile compounds were compared to those of an unpasteurized cider vinegar from France and a vinegar produced in Côte d’Ivoire. The ethanol content for both juices is more or less high with 9.24 ± 0.04 g/L for mango and 12.68 ± 0.39 g/L for papaya. The concentration of acetic acid is the highest of the organic acids for the four vinegars ranging from 37.46 ± 4.6 g/L to 55.85 ± 9.94 g/L. The acetic acid contents of mango and papaya vinegars are close to that of unpasteurized cider vinegar from France but higher than that of vinegar produced in Côte d'Ivoire. Thus, this study allowed the production of "Assabonou" mango and papaya vinegars from two consecutive fermentations (alcoholic then acetic). This process is fast, less expensive and easily applicable. This application case could be an alternative for the processing of seasonal fruits to reduce postharvest losses.

## Graphical abstract

The production of vinegar from mango and papaya was realized through the process of directed alcoholic and acetic fermentation. The concentration of acetic acid is the highest of the organic acids for the four vinegars ranging from 37.46 ± 4.6 g/L to 55.85 ± 9.94 g/L. The acetic acid contents of mango and papaya vinegars are close to that of unpasteurized cider vinegar from France but higher than that of vinegar produced in Côte d'Ivoire.

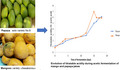

## 1 INTRODUCTION

Every year in Côte d'Ivoire, large quantities of fruit are produced. Some are marketed on the Ivorian territory; others are for export. But a large quantity is lost because many are highly perishable fruits (FIRCA, 2014). Although some of the alternatives for directing consumption have already been forced (jams, fruit concentrates, fruit juices, nectars, purées, etc.), a large quantity of fruit is still left in the fields to rot or to be collected and later disposed of as waste. This is the case with papaya, mango…which are very perishable fruits, where over‐ripe papayas or mangoes are usually discarded and lead to high wastage (Grewal, Tewari & Kalra, 1988). These practices create both an ecological and economic problem. Other alternatives such as processing by fermentation have been proposed and applied in some countries. Most often, the resulting product is fruit wine which is often distilled and has a variable concentration of alcohol. But the impact of these wines is very limited because the alcoholic beverage market is dominated by grape wine and beer and also because consumers are reluctant to try them (Hidalgo et al., 2010). Côte d'Ivoire produces more than 150,000 tons of fresh mangoes each year and exports about 10% of this production to the European market. Postharvest losses are estimated to be about 20%–25% of total production due to pathogen contamination during postharvest handling (Al‐Hindi et al., 2011). Contamination of fruit with fungal infections can occur from the field or during postharvest packing operations, in storage and sometimes after purchase by the consumer (Coleacp, 2011). Their development is favored by the fruit ripening process. These rots affect the market value of the fruit and cause losses of up to 90% (Kouadia et al., 2019).

Like other fruits grown in Côte d'Ivoire, nearly 90% of papaya production is for export. Papaya is very little processed in Côte d'Ivoire (FIRCA, 2014). A better valorization of papaya and mango by tranformation into various end products with a long shelf life will thus contribute to stabilizing the producers' exploitation. It will also make it possible to create new economic activities that create jobs and, consequently, to combat unemployment in Côte d'Ivoire (Soro et al., 2012). Above all, it will make it possible to provide consumers with news product that can satisfy their food needs (Ejemni & Mejri, 2006). The general objective is to set up a protocol for processing certain fruits (mango and papaya) from Côte d'Ivoire into fermented products (wine, vinegar).

## 2 MATERIALS AND METHODS

### 2.1 Material

#### 2.1.1 The vegetal material

**Figure 1:**
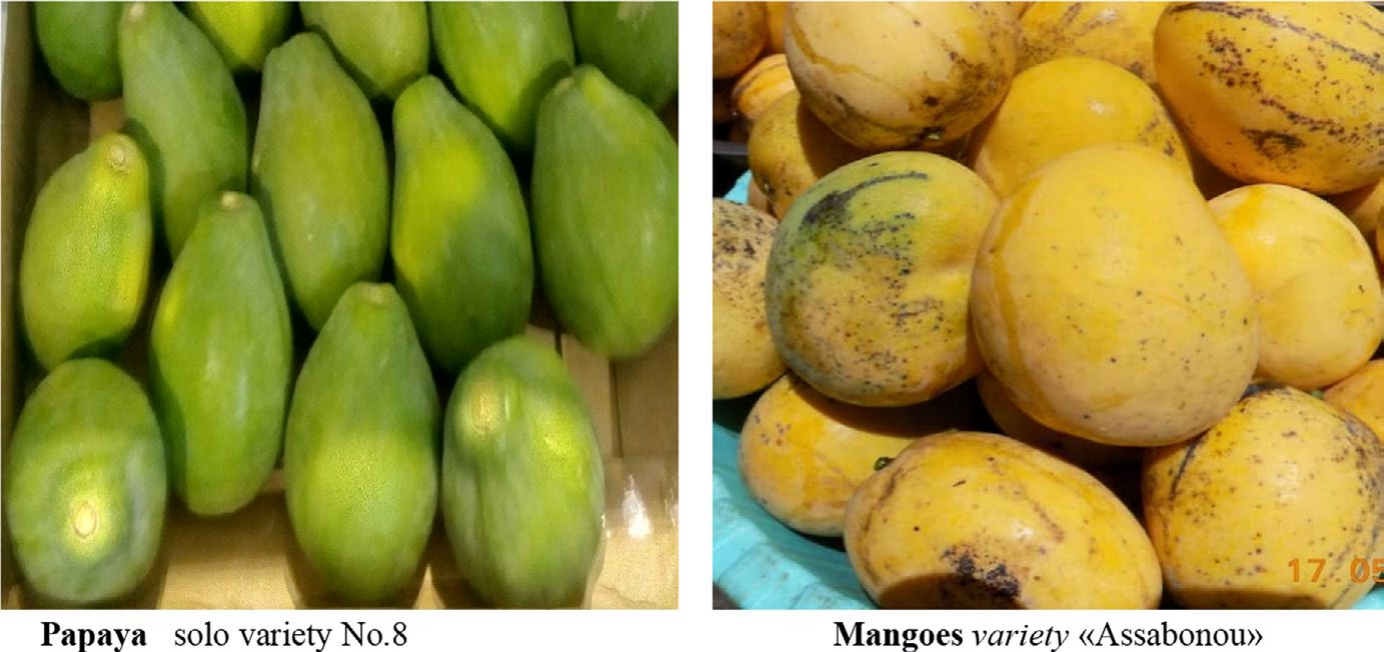
Mangoes (*Mangifera indica) variety* «Assabonou» and Papaya (*Carica papaya)*

The vegetal material consisted of mangoes (*Mangifera indica)* variety «Assabonou» and papaya (*Carica papaya)* solo variety No.8 purchased in the city of Yamoussoukro (Figure 1).

#### 2.1.2 Biological material

The biological material consists of *Saccharomyces cerevisiae* (SAF‐LEVURE baker's yeast, France) and acetic acid bacteria from an unpasteurized apple cider vinegar (Vermont village, France) purchased at the supermarket.

### 2.2 Methods

#### 2.2.1 Sampling

The mangoes and papayas were purchased in the city of Yamoussoukro for ten (10) Kg of each fruit. They were packaged in a perforated cardboard box and then sent to the Microbiology and Food Biotechnology Laboratory of the Nangui Abrogoua University for the various tests.

#### 2.2.2 Juice extraction

The fruits were first sorted according to their degree of ripening. Sorting is a necessary operation for two reasons: either the fruit is too ripe and therefore part of it would be rotting, or the batch could contain immature fruit, which would affect the extraction yield. Then, they were washed and soaked in boiling water for 30 min at 100°C. This operation not only prevents the browning of the finished product, but also allows the partial destruction of the vegetative forms of microorganisms that can alter the product over time. Finally, the fruits were peeled and the pulp was extracted, eliminating the pits for mangoes and the seeds for papayas. The pulp was crushed with a mixer and filtered with muslin. The resulting filtrate was weighed. For five (5) kg of fruit, three liters of juice was obtained for the mango and three and a half liters for the papaya.

#### 2.2.3 Alcoholic fermentation

Before sowing for fermentation, the juices were concentrated in sugar. The initial sugar content of the juices is determined by a refractometer; then, the concentration is carried out by adding a sugar solution (sucrose powder) to reach a refractometric dry extract of 18° Brix. The pH of the fruit juice was also adjusted to 4 by adding lemon juice. The resulting juices packaged in hermetically sealed bottles were immersed in boiling water for five minutes and allowed to cool to a temperature of 30°C. These pasteurized fruit juices were inoculated with 1% baker's yeast (SAf‐Levure). The fermentation of the juices was carried out in three trials in 500 ml vials at the temperature of the experiment room (25–30°C). The refractometric dry extract (RDE) is measured daily until stabilization, marking the end of the alcoholic fermentation. At the beginning and at the end of fermentation, aliquots of juice were taken for various physico‐chemical analyses.

#### 2.2.4 Acetous Fermentation

##### Preparation of the inoculum

A culture medium for acetic bacteria has been prepared. It consists of yeast extract, MgSO4, glucose, NH4 + PO4, sodium nitrate and mannitol. 100 ml has been prepared. After sterilization, 50 µl acetic acid and 300 µl alcohol were added. 100 ml was dispensed into test tubes at a rate of 10 ml per tube. To the contents of each tube was added 1 ml of unpasteurized vinegar. The test tubes were incubated in a water bath at 35°C for one week. After one week, the optical density of each culture medium was read.

##### Fermentation

After alcoholic fermentation, the alcoholic beverages obtained were centrifuged for 10 min at 8,500 g in a refrigerated centrifuge (SIGMA 3–15 p). The supernatant was collected under aseptic conditions and inoculated with the previously prepared acetic strain at a content of 1%, that is, approximately 10^9^ CFU/ml. The drinks thus inoculated were placed in the Bain‐Marie at 30°C and manually stirred daily. The titratable acidity was also monitored during the acetic fermentation until stability was reached, marking the end of the acetic fermentation. Aliquots were also taken at the end of fermentation for analysis of organic acids and volatile compounds. Vinegars sold in Côte d'Ivoire and France were used as controls.

#### 2.2.5 Determination of titratable acidity

Total titratable acidity was determined using the standard method described by Amoa‐Awua et al. (1996) on 5 ml of juice fermented. Total titratable acidity was determined by titrating samples against 0.5 N NaOH using two to three drops of 1% phenolphthalein as indicator. The total titratable acidity was calculated as a percentage of acetic acid using the relationship:

$$\begin{array}{c} \text{\%Titratable acidity}=\text{Vol(NaOH)}\times \text{N(NaOH)}\times 0.06\times 100÷\text{PE}\\ \text{Vol(NaOH)}=\text{Volume of sodium hydroxide solution}\\ \text{N(NaOH)}=\text{Normality of the sodium hydroxide solution}\\ \text{PE}=\text{Testsample}\\ 0.06=\text{milliequivalent grams of acetic acid} \end{array}$$

#### 2.2.6 Organic acids and volatile compounds determination

##### Samples preparation

Samples were centrifuged at 4,000 rpm for 20 min, and supernatants were filtered through a 0.20 mm Millipore membrane filter (Sartorius AG) and then stored at −20 C until analysis.

##### Organic acids content

Organic acid was determined by high‐performance liquid chromatography (HPLC) as previously described by N’guessan (2009). Analyses were carried out with an ion‐exclusion ORH‐801 column (300 × 6.5 mm) (Interchrom) preceded by a Universal Guard CartritgeHolder column. The high‐performance liquid chromatograph system (LC‐6A, Shimadzu Corporation, Japan) was equipped of a Shimadzu LC‐6A pump. Column effluents were monitored by a UV detector (SPD‐6A, Shimadzu Corporation) set at 210 nm. The mobile phase (0.004 N H2SO4) used at a low rate of 0.8 ml/min was filtered through a 0.45 mm Millipore membrane filter (Sartorius AG, Goëttingen). A 20 μl injection volume was used for HPLC samples, and the analyses were done in duplicate. The organic acids standards were dissolved in distilled water at concentrations ranging to 0.05 to 0.4 g/L, filtered, and injected as the samples. Organic acids were identified and quantified by comparison of their retention times and peak areas with those of standards.

##### Ethanol and volatile compounds content

Ethanol and volatile compounds were determined by Gas Chromatography using a Shimadzu CG‐14A (Tokyo, Japan) gas chromatograph equipped with a Porapak Q 100 /120 glass column. The injector and detector are at temperatures of 200°C and 250°C, respectively. Helium with a flow rate of 2 kg/cm^2^ was used as carrier gas. An aliquot of 2.0 μl of the sample treated as above was injected into the chromatograph. The identification of the chromatogram peaks was carried out by comparison with the retention time of the corresponding standards, while the percentage of each identified compound in the sample was determined by comparison with the areas of the standards.

### 2.3 Statistical analysis

Software R. 3‐01, ANOVA method with Duncan's post hoc test, significance level 5% was used to calculate the means, the standard deviations of parameters analyzed. This software also made it possible to compare the means of parameters of the samples and to determine whether the differences observed in the means of the parameters are significant at the 5% threshold.

## 3 RESULTS AND DISCUSSION

**Figure 2:**
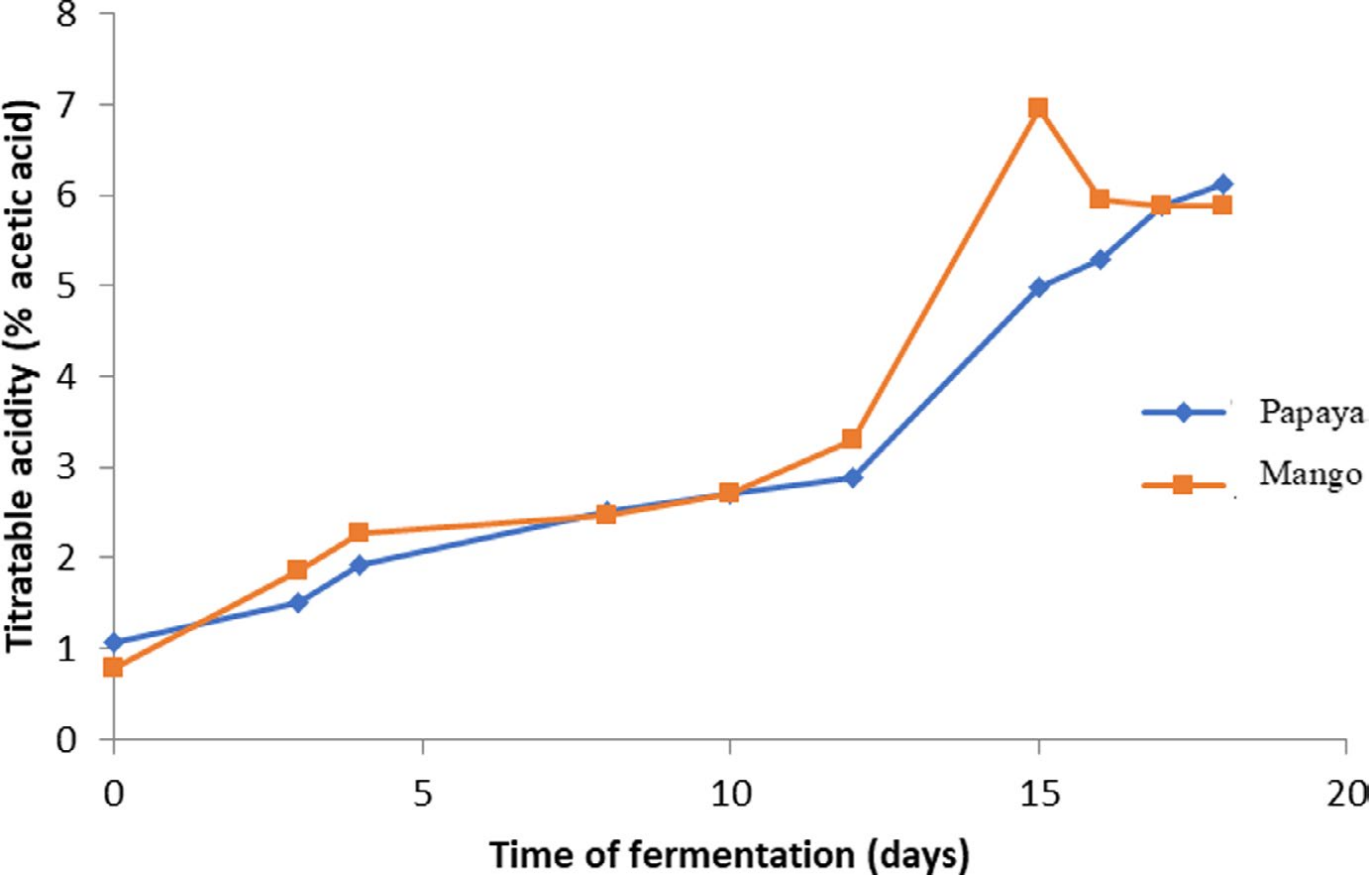
Evolution of titratable acidity during acetic fermentation of mango and papaya juices

This study was carried out with a view to valorizing seasonal fruits with high perishability through the production of organic vinegars following a double fermentation. The first fermentation is an alcoholic fermentation of the fruit juices carried out by the yeast *Saccharomyces cerevisiae* followed by an acetic fermentation of the fruit juices carried out with strains of acetic bacteria from unpasteurized cider vinegar. The juices of the local variety of mango "assabonou" and papaya "solo no. 8" were used as the basic substrate. Alcoholic fermentation was batch fermentation. Thus, ethanol which is the volatile compound mainly produced during alcoholic fermentation of mango and papaya juices was of 12.69 ± 0.39 g/L in papaya juice and 9.25 ± 0.04 g/L in mango juice. These ethanol contents obtained in the two juices are significantly different (*p* < .05) (Table 1). These results are consistent with the work of Ejemni and Mejri (2006) and Silva et al. (2007) who showed ethanol production during alcoholic fermentation of dates and cashew apple. Indeed, ethanol is the essential product of the metabolism of yeasts which transform fermentable sugars into alcohol under anaerobic conditions. This indicates the effectiveness of yeast (*Saccharomyces cerevisae*) metabolism. During acetic fermentation, the titratable acidity level increases in both juices. However, after 15 days of fermentation, a decrease in acidity is observed in the mango juice. At the end of acetic fermentation, mango and papaya vinegars have a total acidity of 6.12 ± 0,14% and 5.88 ± 0,42%, respectively. Acidities rate obtained in juices are not significantly different (P ˃ 0.05) (Figure 2). These levels of acidity are in accordance with the European standard, which should not be less than 5% (SNFV, 2002). The increase titratable acidity observed during fermentation is probably due to the accumulation of organic acids, mainly acetic acid produced by the acetic acid bacteria that constitute the dominant microflora of this fermentation but also Yeast has been reported to produce small amount of acetic acid as by‐products (Chidi et al., 2015; Ferreira et al., 2006) during alcoholic fermentation. Indeed, this acetic flora, notably *Acetobacter aceti, Gluconobacter oxydans,* and *Gluconoacetobacter xylinum,* can develop in symbiosis with certain yeasts such as *Saccharomyces cerevisae, schizosaccharomyces pombe, Brettanomyces bruxellensis, Torulaspora delbrueckii* in certain beverages to produce vinegar (Teoh et al., 2004). After this increase, it was found that the acetic acid content was reduced over time in mango juice. Acetic acid is a compound that evaporates easily. This influenced loss of acetic acid through evaporation when exposed to air (Sanarico et al., 2003). To assess the quality of the vinegars produced, their profile and their composition in organic acid and volatile compounds compared to that of an unpasteurized cider vinegar from France and a vinegar produced in Côte d’Ivoire were compared. High‐performance liquid chromatography (HPLC) detected five (5) organic acids (tannic acid, oxalic acid, citric acid, lactic acid, acetic acid) in mango and papaya vinegars compared to four (4) in France and two (2) in Côte d'Ivoire (Table 2). The four (4) organic acids detected in the unpasteurized cider vinegar from France which are tannic, citric, lactic, and acetic acids are among of the 5 organic acids of Assabonou mango and papaya vinegars solo. The acetic and tannic acids, organic acids present in all the vinegars analyzed, are the only organic acids detected in the vinegar produced in Côte d’Ivoire. The increase in the level of acids in mango and papaya vinegars during acetic fermentation indicates the metabolism of acetic bacteria that are capable of oxidizing ethanol into acetic acid in an aerobic environment. Alcohol is therefore a source of carbon for acetic bacteria. The analysis of the four vinegars revealed acetic acid concentrations ranging from 37.46 ± 4.6g/L to 55.855 ± 9.94 g/L. The maximum concentration of acetic acid is observed with the unpasteurized cider vinegar from France. The acetic acid concentrations of papaya vinegar (54.53 ± 0.38 g/L) and mango vinegar (53.44 ± 1.49 g/L) are not significantly different (P ˃ 0.05) to those of unpasteurized cider vinegar from France (Table 2). Similarly, these contents are higher than those of the work of Ejemni and Mejri (2006) who obtained an acetic acid concentration of 48.9 g/L in date waste vinegar. Vinegar is a very excellent antiseptic because it contains acetic acid, which has a bacteriostatic effect on certain groups of pathogenic bacteria. (Benaoun, 2007). It should therefore be noted that the high acetic acid content of mango and papaya vinegars makes these vinegars products of acceptable hygienic quality. The production of acetic acid in "assabonou" mango and "solo n°8" papaya vinegars is accompanied by a more or less high concentration of lactic acid. In addition, vinegars obtained from papaya and mango contain, respectively, 9.13 ± 1.63 g/L and 8.10 ± 1.31 g/L of citric acid against 5.15 ± 3.28 g/L of citric acid in France vinegar. The rate of citric acid in the vinegar obtained from papaya is not significantly different to that obtained from mango (P ˃ 0.05). But the rates of citric acid in these two vinegars are significantly different (P ˂ 0.05) to the citric acid obtained in the vinegar of France. (Table 2). The presence of these organic acids makes mango and papaya vinegars high value‐added products. Indeed, the acrid and pungent character of acetic acid is reduced in mango and papaya vinegars by the high content of lactic acid and the presence of other organic acids (citric acid, tannic acid) as previously reported (Anonyme^1^, ^2015^). In addition, citric acid is an essential component of the cell's energy metabolism, formed mainly from glucose. It also comes from other metabolic pathways and from food. It is an organic acid that is used by the kidney to maintain the acid–base balance (Anonyme^1^, ^2015^). Similarly, lactic acid is an energy intermediate and produces half of the energy (18 ATP molecules) that glucose is capable of producing (Cazorla et al., 2001). Lactic acid is therefore first and foremost an essential substrate for aerobic metabolism by playing a central regulatory role in the cell and throughout the body (Gladden, 2004). Thus, the presence of these organic acids in "assabonou" mango and "solo n°8" papaya vinegars gives them nutritional characteristics of the fruits from which they are produced. In addition, mango and papaya vinegars contain volatile compounds that are not present in the other types of vinegar analyzed during this study. Indeed, gas chromatography allowed the detection of six (6) volatile compounds in mango and papaya vinegars against three (3) in vinegar from France and three (3) in vinegar from Côte d'Ivoire (Table 3). The three (3) volatile compounds detected in the vinegars of France and Côte d'Ivoire, namely ethanol, propanol‐1, and propanol‐2, are among the five (5) volatile compounds detected in the vinegars of mango *assabonou* and papaya solo. n°8. However, the level of ethanol is higher in *assabonou* mango vinegar (0.308 ± 0.017g/L) than other vinegars (Table 3). These results are in agreement with European regulations that set the residual alcohol content below 0.5% (SNFV, 2002).

**Table 1:** Composition and concentration of volatile compounds in mango and papaya juices after alcoholic fermentation

Volatile Compounds (g/L)	Fermented juices	
	Papaya	Mango
Methanol	26.59 ± 1.84 × 10^−5a^	0^b^
Acetaldehyde	5.74 ± 10^−5a^	11.46 ± 4.90 × 10^−5b^
Ethanol	12.69 ± 0.39^a^	9.25 ± 0.04^b^
Propanol 1	25.16 ± 3.90 × 10^–5^	0.00009 ± 3 ×10^−5b^
Ethyl acetate	1.97 ± 2.79 × 10^–5^	13.23 ± 18.71 × 10^–5^

Note

On a line, means values carrying different alphabetical letters are statistically different to the level of 5% (Duncan's multiple test range at *p* < .05).

**Table 2:** Concentrations of organic acids in vinegar products

	Control vinegars		Vinegars products	
Organic acids (g/L)	VIC	VIF	VM	VP
Tannic acid	0.057 ± 5.9 × 10^−5a^	0.256 ± 0.09^b^	0.16 ± 0.09^c^	0.12 ± 0.01^c^
Oxalic acid	0^a^	0^a^	0.12 ± 0.13^b^	0.15 ± 0.02^b^
Citric acid	0^a^	5.15 ± 3.28^b^	8.10 ± 1.31^c^	9.13 ± 1.63^c^
Lactic acid	0^a^	8.59 ± 5.18^b^	89.80 ± 0.26^c^	32.53 ± 0.76^d^
Acetic acid	37.46 ± 4.6^a^	55.85 ± 9.94^b^	53.44 ± 1.49^c^	54.53 ± 0.38^bc^

Note

On a line, means values carrying different alphabetical letters are statistically different to the level of 5% (Duncan's multiple test range at *p* < .05). VIC = industrial vinegar from Côte d'Ivoire; VIF = industrial vinegar from France; VM = vinegar obtained from mango; VP = vinegar obtained from papaya.

**Table 3:** Concentrations of Volatile compounds in vinegar products

Volatile compounds ^(g/L)^	Control vinegars		Vinegars products	
	VIC	VIF	VM	VP
Methanol	0^a^	0^a^	0^a^	8.48 ± 3.78 × 10^−5b^
Acetaldehyde	0^a^	0^a^	39.59 ± 7.02 × 10^−5b^	4.146 ± 5.86 × 10^−5c^
Ethanol	0.025 ± 0.011^a^	0.052 ± 0.001^a^	0.308 ± 0.017^b^	0.006 ± 9×10^−4c^
Propanol‐2	3.44 ± 1.72 × 10^−5a^	4.18 ± 4.18 × 10^−5a^	22.059 ± 8.42 × 10^−5b^	25.76 ± 16.7 × 10^−5b^
Propanol‐1	0.011 ± 0.01^a^	0.018 ± 0.001^ab^	0.022 ± 10^−3b^	0.019 ± 89.8 × 10^−5ab^
Ethyl acetate	0^a^	0^a^	15.23 ± 2.12 × 10^−5b^	0^a^

Note

On a line, means values carrying different alphabetical letters are statistically different to the level of 5% (Duncan's multiple test range at *p* < .05). VIC = industrial vinegar from Côte d'Ivoire; VIF = industrial vinegar from France; VM = vinegar obtained from mango; VP = vinegar obtained from papaya.

## 4 CONCLUSION

The transformation of "assabonou" mango and "solo°8" papaya into vinegar by double fermentation (alcoholic and acetic) showed a production of ethanol during alcoholic fermentation as well as considerable production of acetic acid and other organic acids during acetic fermentation. Fermentation has made it possible to obtain "assabonou" mango and "solo n°8" papaya vinegars of good nutritional quality proved by the presence of organic acids necessary for the metabolism of the cell and therefore for the maintenance of human health. The composition of these vinegars respects the European standards and regulations concerning vinegars. The production of vinegar on a large scale from mangoes and papayas but especially from seasonal fruits in Côte d’Ivoire could be considered and would present a very interesting solution to reduce postharvest losses. It would help to put a new product specific to our agriculture on the national and international market.

## CONFLICT OF INTEREST

Authors have no conflict of interest regarding the publication of paper.

## Consent for Publication

All listed authors have read the final manuscript and provided consent for publication.

## Ethical Review

“This study does not involve any human or animal testing.”

## Data Availability

The data files associated with this study have been submitted along with this manuscript and are available upon request. Please contact the Corresponding Author with any questions or concerns.
